# Integrating *In Silico* Prediction Methods, Molecular Docking, and Molecular Dynamics Simulation to Predict the Impact of ALK Missense Mutations in Structural Perspective

**DOI:** 10.1155/2014/895831

**Published:** 2014-06-26

**Authors:** C. George Priya Doss, Chiranjib Chakraborty, Luonan Chen, Hailong Zhu

**Affiliations:** ^1^Medical Biotechnology Division, School of Biosciences and Technology, VIT University, Vellore, Tamil Nadu 632014, India; ^2^Department of Bio-Informatics, School of Computer and Information Sciences, Galgotias University, India; ^3^Key Laboratory of Systems Biology, Shanghai Institutes of Biological Sciences, Chinese Academy of Sciences, China; ^4^Department of Computer Sciences, Hong Kong Baptist University, Kowloon Tong, Hong Kong

## Abstract

Over the past decade, advancements in next generation sequencing technology have placed personalized genomic medicine upon horizon. Understanding the likelihood of disease causing mutations in complex diseases as pathogenic or neutral remains as a major task and even impossible in the structural context because of its time consuming and expensive experiments. Among the various diseases causing mutations, single nucleotide polymorphisms (SNPs) play a vital role in defining individual's susceptibility to disease and drug response. Understanding the genotype-phenotype relationship through SNPs is the first and most important step in drug research and development. Detailed understanding of the effect of SNPs on patient drug response is a key factor in the establishment of personalized medicine. In this paper, we represent a computational pipeline in anaplastic lymphoma kinase (ALK) for SNP-centred study by the application of *in silico* prediction methods, molecular docking, and molecular dynamics simulation approaches. Combination of computational methods provides a way in understanding the impact of deleterious mutations in altering the protein drug targets and eventually leading to variable patient's drug response. We hope this rapid and cost effective pipeline will also serve as a bridge to connect the clinicians and *in silico* resources in tailoring treatments to the patients' specific genotype.

## 1. Introduction

The vast amounts of data available from whole genome sequencing represent a challenge in the analysis, often requiring automated methods for annotation and prioritization of the variants. In attaining this milestone, profiling the most common single nucleotide polymorphisms (SNPs) by computational approach became powerful and inexpensive enough to jumpstart the personalized genomics area [1–4]. SNPs are not only considered as markers in constructing genetic maps but also have the potential as direct functional polymorphic variants involved within complex diseases, as well as drug response. A majority of the nonsynonymous SNPs (nsSNPs) associated with human disorders are caused by alteration in structural stability [5, 6] or based on attractive notion that these mutations directly disrupt the ligand interactions sites [7–9]. The last decade has witnessed a tremendous increase in the number of studies comprehensively in understanding the genetic basis for interindividual drug response variability [10, 11]. Understanding the underlying mechanisms of phenotypic variability in drug response at the protein level is foremost in the establishment of personalized medicine [12]. There is an urgent need to categorize the functionally important nsSNPs as deleterious or disease causing ones in a cost-efficient manner. More sophisticated fast and cost effective* in silico* prediction methods are developed to screen the degree of deleteriousness (affect protein function) of an nsSNP based on sequence information and structural attributes and made available on the World Wide Web [13–19]. Each algorithm has its own unique strengths and weaknesses [1, 2]. Most of them utilize sequence, structure information, or combination of both along physiochemical properties of amino acids to classify them as pathogenic or neutral (Table 1). Insight into the knowledge of the three-dimensional structure (3D) of a gene product is a key component in determining the impact of a mutation in causing disease. 3D structural information provides valuable information about the environmental changes upon mutation with altered active sites, stability, and flexibility of the protein. More than 88,000 macromolecular structures are deposited in the protein data bank (PDB). To measure the adverse effects of mutation on protein structure, it is necessary to map the mutation and construct 3D models by homology modeling methods. Recent advances in structural genomics, modeling techniques, and drug discovery technologies also make it possible to attempt structural interpretation of drug-target interaction upon mutation and their differential therapeutic responses [11, 20].

ALK, a receptor tyrosine kinase in the insulin receptor superfamily, was initially identified in activated oncogenic fusion forms, most common being the nucleophosmin ALK in anaplastic large-cell lymphomas and paediatric cancer as well as neuroendocrine tumors [21]. ALK consists of 1620 amino acids in which 1030 residues in the extracellular region encompass multiple subdomains including an LDL-A domain (low-density lipoprotein class A domain), a MAM (meprin) domain, and a glycine-rich region. The cytoplasmic portion contains 563 residues and includes the kinase catalytic domain. This full-length form is implicated in malignancies where ALK promotes tumorigenesis via activation by autocrine and paracrine growth-promoting loops involving the putative endogenous ALK ligands PTN (pleiotrophin) and MK (midkine) [22]. Originally, truncated form of ALK was first described in non-Hodgkin's lymphoma with fusion protein product of ALK and nucleophosmin (NPM). Later on additional fusion partners of ALK were identified with reports of variable expression of ALK in adenocarcinomas of the lung, neuroblastomas, breast, and esophageal cancers [23–25]. In familial neuroblastoma, mutations in ALK alter the protein kinase domain (1116–1392), thereby leading to constitutive activation of the receptor kinase and phosphorylation of downstream targets. This results in heightened cell proliferation, invasion, and survival [26, 27]. Mutations in protein kinase domain F1174 and R1275 are the most frequently reported in neuroblastomas [28, 29]. The cells harboring the F1174 and R1275 mutations in ALK proved to be more sensitive towards small molecule inhibitors [28, 30]. Most recently crizotinib was approved for use in non-small-cell lung carcinoma (NSCLC) patients as ALK inhibitor [3, 30]. Unfortunately, cancers have eventually developed resistance to crizotinib. In 2010, Choi et al. [31] identified two secondary mutations (C1156Y and L1196M) within the kinase domain of ALK fusion protein, which confer marked crizotinib resistance.

**Figure 1:**
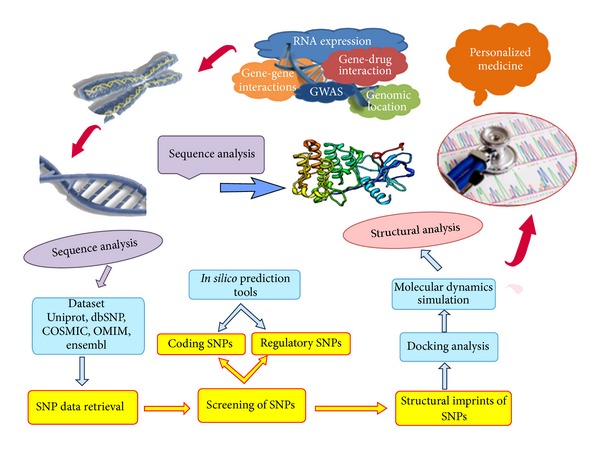
Flow chart for the proposed methodology.

Till date, no combinatorial approach was undertaken in ALK with the interplay of* in silico* prediction methods along with molecular docking and molecular dynamics. This insisted us to design* in silico* framework in the discovery of new drugs or drug targets in ALK owing to the changes brought by deleterious nsSNPs (Figure 1). As an initial step, we listed out the deleterious nsSNPs in ALK based on sequence-structural-based algorithms prediction scores of SIFT [13], PolyPhen 2.0 [14], I Mutant 3.0 [15], SNAP [16], SNPs&GO [17], PhD-SNP [18], and Align GVGD [19]. In the next step, we conducted* in silico* functional analysis to identify the SNPs associated with regulatory mechanisms [32] and posttranslational modification (PTM). Furthermore,* in silico* mutational analysis was initiated by mapping the deleterious mutations onto the available 3D structure with the help of Swiss-PdbViewer [33]. An atomic level look at the protein dynamics behavior was performed using molecular dynamics simulation [34, 35] to reveal the impact of these mutations on protein structure. Lastly binding affinity between the crizotinib and the deleterious mutations was observed with the aid of molecular docking study [36, 37]. We propose that these findings could provide valuable hints in disease diagnosis and treatment towards personalized medicine.

## 2. Materials and Methods

### 2.1. Dataset Used for SNP Annotation

Human* ALK* gene information data was collected from Online Mendelian Inheritance in Man (OMIM) [38] and Entrez Gene on National Centre for Biological Information (NCBI). The SNP information (protein accession number (NP), mRNA accession number (NM), and SNP ID) was retrieved from dbSNP [39] and UniProt databases [40]. Protein 3D structure was obtained from protein data bank (PDB) [41].

### 2.2. Predicting Functional Context of Missense Mutation

The functional context of nsSNPs in the coding region was predicted using SIFT, PolyPhen 2, SNAP, SNPs&GO, and PhD-SNP. SIFT provides the tolerance index score ranging from 0 (deleterious) to 1 (neutral) of a particular amino acid substitution to protein function based on the sequence alignments. PolyPhen 2 utilizes straightforward physical and evolutionary comparative considerations to predict amino acid substitutions on protein structure and function. PolyPhen 2 calculates and computes the difference in the PSIC (position-specific independent count) score of the two variants. The probabilistic score ranges from 0 (neutral) to 1 (deleterious), and functional significance is categorized into benign (0.00–0.14), possibly damaging (0.15–0.84), and probably damaging (0.85–1). PhD-SNP is a single sequence SVM method (SVM-sequence) that discriminates disease-related mutations from neutral ones based on the local sequence environment of a mutation. SNPs&GO is an SVM-based method which utilizes functional gene ontology (GO) terms to predict the disease-associated mutations from the protein sequence and evolutionary information. Neural network-based screening for nonacceptable polymorphisms (SNAP) utilizes sequence information (secondary structure, solvent accessibility), flexibility, functional effects, and conservation information from various resources to predict the functional effect of each nsSNP in a protein sequence as neutral or nonneutral and provides the information about the estimation about the prediction reliability.

### 2.3. Predicting Protein Stability upon Mutation

Stability change of a protein can be measured by computing the change in its Gibbs-free energy upon folding. Substitution of single amino acid in a protein sequence can result in a significant change in the protein's stability (ΔΔ*G*); positive ΔΔ*G* represents a destabilizing effect and the negative value represents a stabilizing effect on mutation. We employed I-Mutant 3.0 built by unsupervised classification using support vector machine and trained on the most comprehensive dataset derived from ProTherm [42] for the prediction of protein stability change for nsSNPs. The energy difference between native and the mutant protein was calculated based on Gibbs-free energy value, and the predicted free energy change was denoted by DDG value.

### 2.4. Biophysical Characterization of Altered Amino Acid

Assessment of the putative deleterious effect of ALK variants was also performed with the evolutionary conservation method Align Grantham Variation Grantham Deviation (Align-GVGD). To predict the impact of mutations Align-GVGD combines the biophysical characteristic of each altered amino acid and protein multiple sequence alignments generated by T-coffee to classify the scores into seven categories C0, C15, C25, C35, C45, C55, and C65. The variant with the score of C0 is designated as least likely to be deleterious and C65 as most likely to be deleterious.

### 2.5. Functional Characterization of SNPs

The prediction of the phenotypic risks and putative functional effects of a given variant in the regulatory region were assessed using function analysis and selection tool for single nucleotide polymorphism (FASTSNP) [32]. We submitted input in the query form of “Candidate gene” and selected the SNPs for prioritization. FASTSNP utilizes eleven external web servers to designate the twelve phenotypic risks and functional effects along with the ranking system ranging from 0 (no effect) to 5 (very high risk) for each SNP located in the coding 5′, 3, and intronic region. We employed GPS 2.1 [43], NetNGlyc [44], and NetOGlyc 3.1 [45] to predict the functional significance of SNP in phosphorylation, N-glycosylation, and O-glycosylation sites. In GPS 2.1 version, we selected medium level threshold to identify the phosphorylation sites. NetNGlyc 1.0 server utilizes artificial neural networks to scan the sequence information of Asn-Xaa-Ser/Thr sequences. Meanwhile, NetOGlyc server uses neural network-based method to predict the mucin type GalNAc O-glycosylation sites in mammalian proteins.

### 2.6. *In Silico* Mutation Analysis

The crystal structure of human ALK was obtained from the PDB (ID: 3L9P) to generate the starting models for the simulation [46]. Missing loops residues 1117–1122 were modelled using Falc loop [47]. We mapped the deleterious mutations ALK F1174L and ALK R1275Q to their corresponding positions in the crystal structure and mutated the proteins using Swiss-PdbViewer. After mutation, the structure was subjected to optimization and energy-minimized using GROMACS force field. Four different simulations were carried out that include the native type and three mutated models.

### 2.7. Molecular Docking

We utilized AutoDock (V. 4.0) [36] and Patchdock [37] for our molecular docking study. This provides valuable insight into the interaction between the native and mutant proteins of ALK with ligand crizotinib. The structure of ligand crizotinib was generated from smile strings followed by energy minimization. Similarly, we obtained the crystal structure of ALK (PDB ID: 3L9P) with resolution 1.80 Å from the protein data bank. AutoDock program with the Lamarckian genetic algorithms (LGA) was used to perform docking experiments. The Lamarckian GA parameters used in the analysis consist of 30 independent runs, population size of 150, a maximum number of 25,000,000 energy evaluations, number of generation 27,000, mutation rate of 0.02, and a crossover rate of 0.8. Docking was carried out with the grid size of 60, 60, and 60 along the *X*-, *Y*-, and *Z*-axis with 0.375 Å spacing. RMS cluster tolerance was set to 2.0 Å. In order to increase the accuracy of the docked poses by AUTODOCK, we performed docking analysis using PatchDock program. This uses molecular docking algorithm based on structure geometry. The PatchDock algorithm divides the Connolly dot surface representation of the protein molecules into three classes, namely, convex, concave, and flat patches [48, 49]. Then, complementary patches were matched to generate the candidate transformations. Each of the candidate transformation is additionally evaluated by a scoring function which considers both the atomic desolvation energy and geometric fit [50]. Next, root mean square deviation (RMSD) clustering is applied to the candidate solutions to discard redundant solutions. The input parameters for the docking are the PDB coordinate file of the protein and ligand molecule. Three major steps are followed in the PatchDock analysis: (i) surface patch matching, (ii) molecular shape representation, and (iii) filtering and scoring.

### 2.8. Molecular Dynamics Simulation

MD simulation of the complex was carried out with the GROMACS 4.5.4 package using the GROMOS96 43a1 force field [51]. The lowest binding energy (most negative) docking conformation generated by AutoDock was taken as initial conformation for MD simulation. The topology parameters of proteins were created by using the Gromacs program. The topology parameters of crizotinib were built by the Dundee PRODRG server [52]. The complex was immersed in an octahedron box of simple point charge (SPC) water molecules. Eight Na^+^counter-ions were added by replacing water molecules to ensure the overall charge neutrality of the simulated system. The native and mutant complexes were energy-minimized initially by steepest descent 10,000 steps, followed by conjugate gradient method 10,000 steps. In order to equilibrate the system, the solute was subjected to position-restrained dynamics simulation (NVT and NPT) at 300 K for 300 ps. Finally, the full system was subjected to MD production run at 300 K temperature and 1 bar pressure for 20 000 ps. MD simulations were repeated thrice in order to verify the reproducibility of our study.

### 2.9. Principal Component Analysis

The principal component analysis is a technique that reduces the complexity of the data and extracts the concerted motion in simulations that are essentially correlated and presumably meaningful for biological function. In the principal component analysis, a variance/covariance matrix was constructed from the trajectories after removal of the rotational and translational movements. The calculation of the eigenvectors and eigenvalues and their projection along the first two principal components were carried out using essential dynamics (ED) method [53]. A set of eigenvectors and eigenvalues were identified by diagonalizing the matrix. The movements of the protein in the essential subspace were identified by projecting the Cartesian trajectory coordinates along the most important eigenvectors from the analysis.

### 2.10. Analysis of Molecular Dynamics Trajectory

The trajectory files were analyzed by using g_rms, g_rmsf, and g_sas GROMACS utilities in order to obtain the root-mean-square deviation (RMSD), root-mean square fluctuation (RMSF), and solvent accessibility surface area (SASA). Numbers of distinct intermolecular hydrogen bonds formed during the simulation were calculated using g_h bond utility. Numbers of hydrogen bonds are prominent, when donor-acceptor distance is smaller than 3.9 nm and donor-hydrogen-acceptor angle is larger than 90 nm. The trajectory files of PCA were analyzed through the use of g_covar and g_anaeig of GROMACS utilities in order. The analysis of the secondary structure elements of the protein was performed using the program “do_dssp,” which utilizes the DSSP program [54].

## 3. Results

### 3.1. SNP Annotation

Dataset utilized in functional characterisation of 149 nsSNPs in ALK was retrieved from dbSNP, UniProt, and Ensembl databases. Among 149 nsSNPs, 78 nsSNPs were mapped to the cytoplasmic domain, 50 nsSNPs to the protein kinase domain, and remaining 21 nsSNPs to the ligand binding domain.

### 3.2. Prioritizing Functional SNPs

Numerous* in silico* prediction tools with diverse algorithms were used to characterise the functionally significant nsSNPs in ALK. The performance of computational algorithms in identifying the deleterious or neutral nsSNPs in ALK protein is given in Table S1 (see Table S1 in Supplementary Material available online at http://dx.doi.org/10.1155/2014/895831). SIFT predicted 76 deleterious nsSNPs from a total of 149 missense mutations that could bring about a change in the protein function. PolyPhen 2.0 evaluates the location of the amino acid replacement within the identified functional domains and 3D structures. PolyPhen 2.0 predicted 87 nsSNPs to be damaging. SNAP was used to predict the overall severity of the missense mutation based on neural network and improved machine learning approach. SNAP predicted 60 nsSNPs as nonneutral which could bring about a change in protein function. SNPs&GO and PhD-SNP designated 35 and 36 nsSNPs as disease. I Mutant 3.0 predicted 33 nsSNPs that could change the stability of the protein upon mutation. Align GVGD predicted the functional impact on protein as deleterious 59 nsSNPs. In order to prioritize the most potent nsSNPs associated with ALK, the result obtained from the above* in silico* methods was integrated into a single coherent framework. By comparing the prediction scores obtained from all the six* in silico* tools, 2 nsSNPs at positions ALK F1174L and ALK R1275Q in the coding region were predicted to have a functional effect on protein function and stability. FASTSNP identified the nsSNPs that can influence the cellular and molecular biological function, for example, transcriptional and splicing regulation (Table 2). From our analysis, 11 SNPs in 5 upstream and coding regions were found to have a role in the promoter/regulatory region with a risk level of 1–3 upon nucleotide change.

### 3.3. Docking Analysis

**Figure 2:**
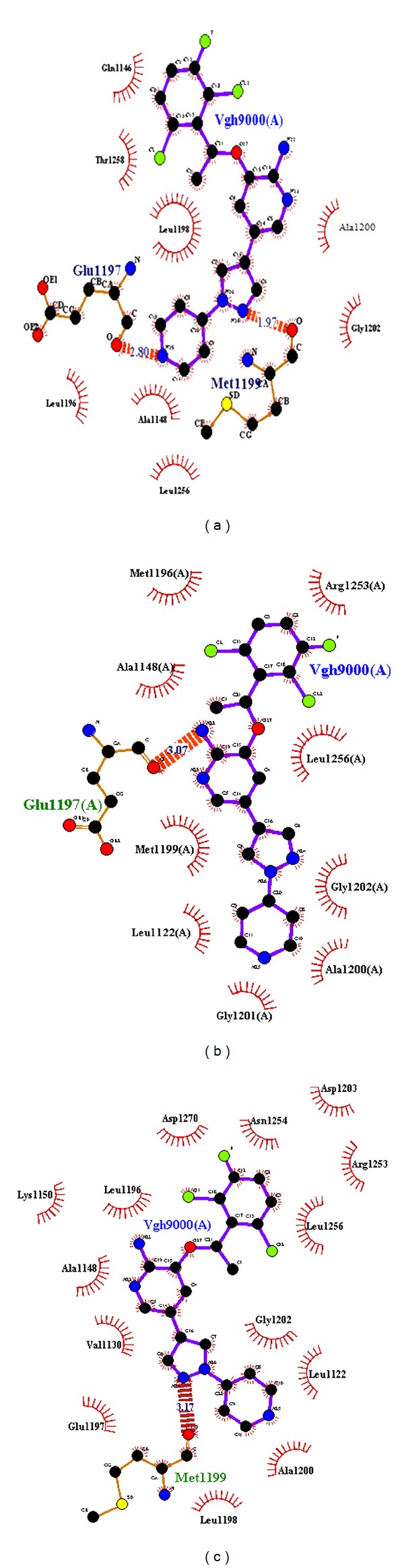
Residual interactions in the protein-drug interface was analyzed by Ligplot (a) Native (b) Mutant F1174L (c) Mutant R1275Q.

To investigate the impact of mutation on the molecular functions of ALK protein, docking analysis was carried out with a specific inhibitor crizotinib. Results indicated that the mutations contribute to weaker interaction with the drug, primarily due to loss of interactions of the drug with surrounding residues. We utilised three complexes, namely, native (ALK-crizotinib), F1174L (ALK F1174L-crizotinib), and R1275Q (ALK R1275Q-crizotinib) for our analysis.  Table 3 displays the lowest calculated binding energy value of ALK docked to the drug. Comparing the binding free energy of ALK to the drug, mutant F1174L exhibited the weakest interaction with the energy value of −7.34 Kcal/mol when compared to the mutant R1275Q (−8.07 Kcal/mol) and native complex (−9.21 Kcal/mol), respectively. Detailed analysis showed that the crizotinib acquired an altered mode of binding in both the mutant complexes. From Figure 2(a), it is clear that in the native model, crizotinib forms hydrogen bond with the active residues M1199 and Q1197 (Table 2). All other contacts were hydrophobic, as noted in the crystal structure [46]. In the mutant F1174L complex, the mutation disrupts the local hydrogen bond between the protein and drug; as a result, only one hydrogen bond is formed (Figure 2(b)), whereas, in mutant R1275Q complex, one hydrogen bond was formed and lost, when compared to the native complex (Figure 2(c)). The change in the hydrophobic contact in native and mutant complexes is elucidated in Table 3. Validation of docking results is needed to optimize the error and uniformity. Docking score and atomic contact energy (ACE) of the native and mutant complexes were calculated using Patch Dock (Table 4). Obtained results confirm that native complex exhibited the highest docking score of 6894 and ACE value of −298.28 when compared to the other two mutant complexes. This result signifies better conjugation of inhibitor to the binding pocket of the receptor. Mutant F1174L complex exhibited the least binding affinity towards crizotinib, which was confirmed by the docking score of 5432 and ACE value of −144.17. These results were in concordance with the results obtained from AutoDock.

### 3.4. Simulation Study of Native and Mutant Complexes

**Figure 3:**
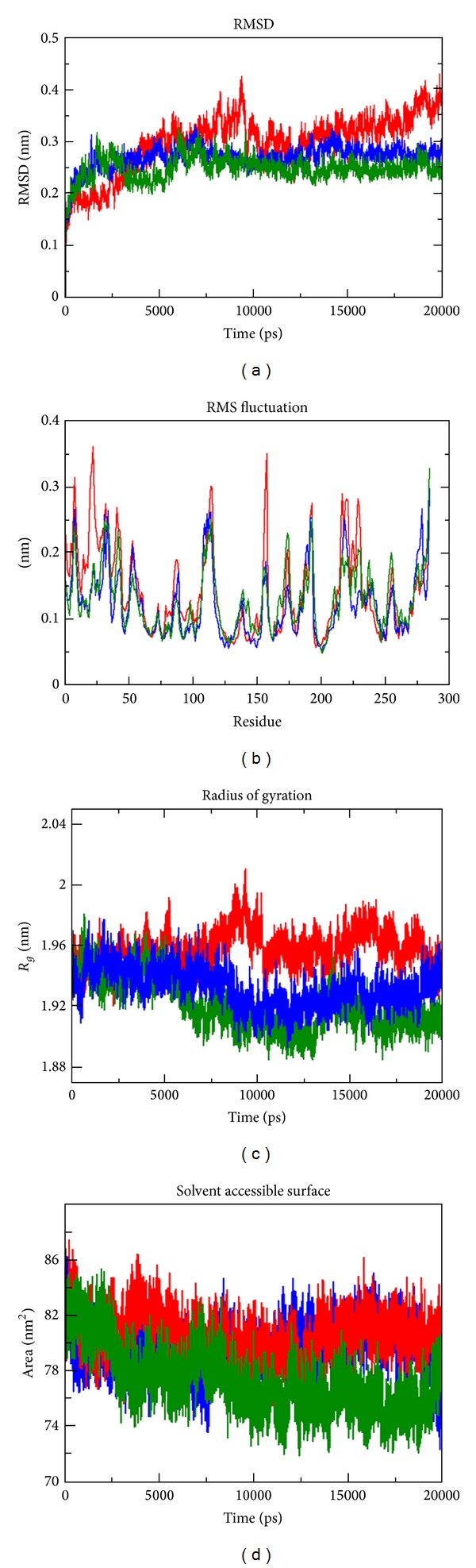
Analysis of RMSD, RMSF, Rg, and SASA of native and mutant ALK-crizotinib complex at 20000 ps. (a) Time evolution of backbone RMSDs of the native and mutant structures. (b) RMSF of the carbon alpha over the entire simulation. The ordinate is RMSF (nm), and the abscissa is residue. (c) Rg of the protein backbone over the entire simulation. The ordinate is Rg (nm), and the abscissa is residue. (d) The ordinate is SASA (nm^2^), and the abscissa is time (ns). The symbol coding scheme is as follows: native (green colour), mutant F1174L (red colour), and R1275Q (blue colour).

**Figure 4:**
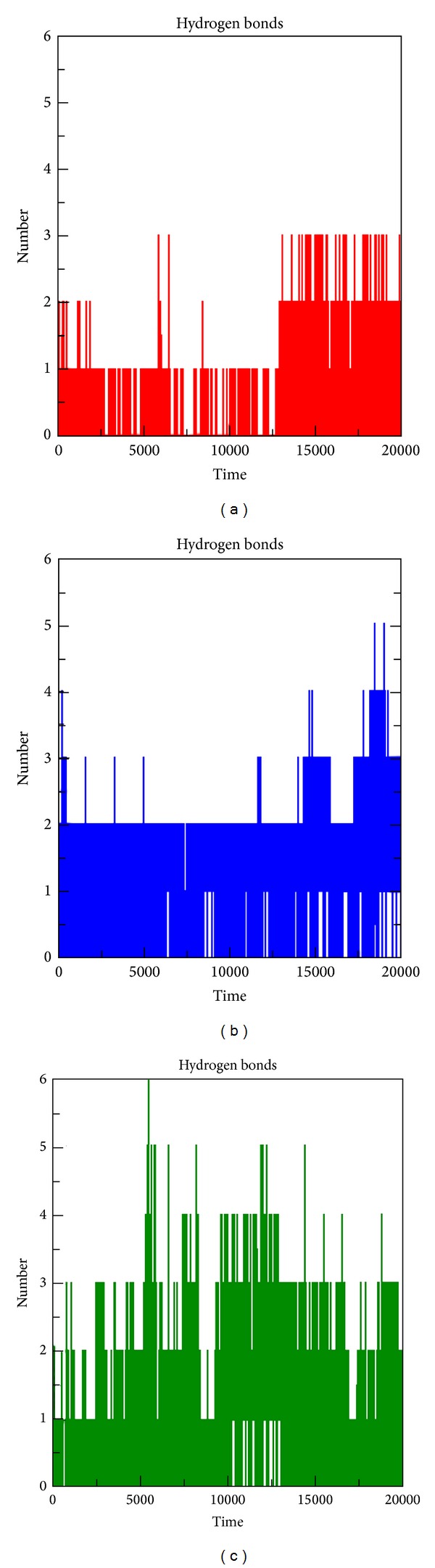
Analysis of intermolecular NH bond of native and mutant ALK-crizotinib complex at 20000 ps. Average number of intermolecular hydrogen bonds in native and mutant versus time. (a) Native, (b) mutant F1174L, and (c) mutant R1275Q.

**Figure 5:**
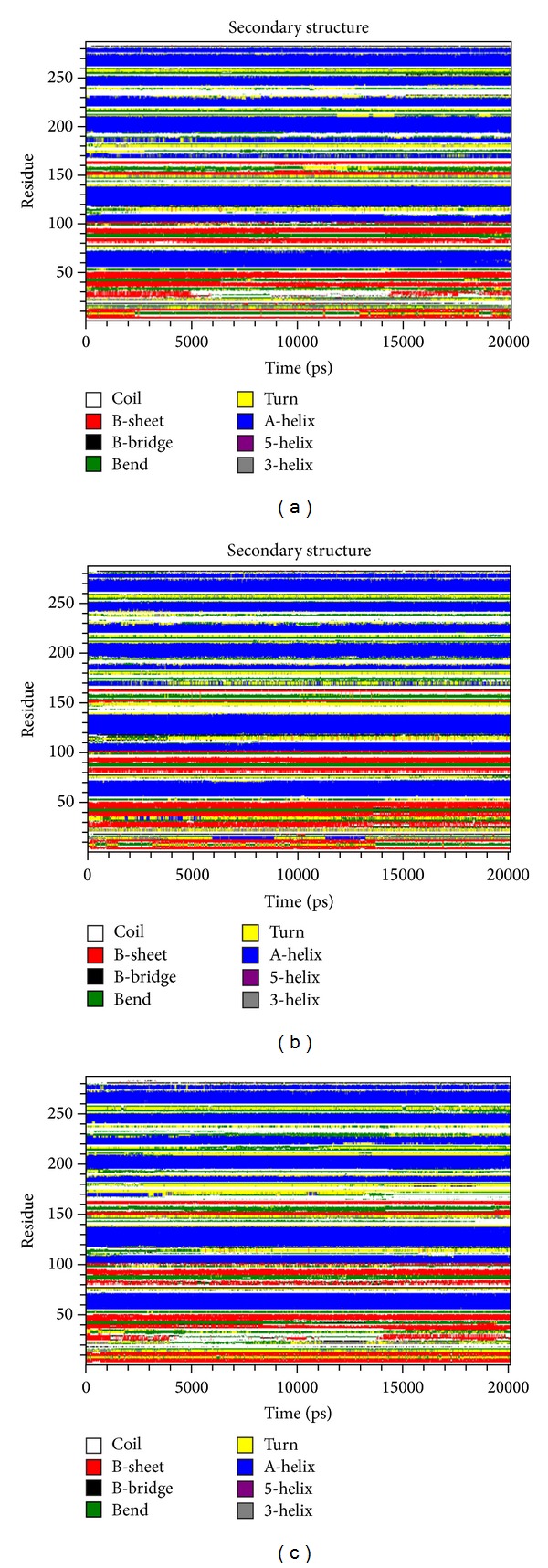
Time evolution of the secondary structural elements of the protein at 300 k (DSSP classification). (a) Native, (b) mutant F1174, and (c) mutant R1275Q.

The results obtained from the above docking analysis provoked us to explore the dynamic behaviour of native and mutant complexes. We analysed the root mean square deviation (RMSD), root mean square fluctuation (RMSF), radius of gyration (Rg), solvent accessible surface area (SASA), number of hydrogen bonds (NH), and secondary structure variation between the native and mutant (F1174L and R1275Q) complexes. Three independent simulations were carried out for the native and mutant complexes for a total of simulation time. We found that no significant drift occurred in amino acid trajectories initiated from the native and mutant complexes of three independent MD simulations. For all three simulations, the protein structures could be aligned with C*α*-RMSD below 3.2 Å (Figure S1). Indeed, native and mutant R1275Q complexes tend to reach a steady equilibrium, while RMSD of the mutant complex F1174L was noticeably high. Mutant complex F1174L remained distinguished throughout the simulation resulting in maximum backbone RMSD of ~0.41 nm. This difference in the deviation range explains the change in stability of the mutant protein, which in turn reflects the impact of substituted amino acid in the protein structure. The magnitude of fluctuation together with a small deviation in average RMSD after the relaxation period leads to the conclusion that the simulation produced stable trajectory, thus providing a suitable basis for further analysis (Figure 3(a)). As well as change in conformation, flexibility of the structure can be altered by mutations. We calculated the C*α*-RMSF to measure overall flexibility of the native and mutant complexes. Mutant F1174L complex shows a conformational change in the protein structure (as indicated in backbone RMSD) with increase in the C*α*-RMSF being also observed. This suggests that F1174L mutation affects the binding of crizotinib and makes the backbone more flexible to move. It is to be noticed that R1275Q mutation affects the neighbouring residues at the maximum of ~0.46 nm fluctuation indicating a gain of flexibility due to mutation (Figure 3(b)). Furthermore, the flexibility of mutant R1275Q was found to be in consistent with the native ALK. This might be due to the restriction caused by the surrounding residues in the active site of protein due to mutation in ALK protein. Overall, results suggest that there exists a significant change of structural deviation in the mutant complex F1174L when compared to the native. To measure the compactness of the hydrophobic core, solvent-accessible surface area (SASA) was monitored throughout all simulations. Both the mutant complexes indicate greater values of SASA (~87 nm^2^) with time when compared to the native protein (Figure 3(c)). A major contributor to the increased exposure was the loss of hydrophobic contacts between the residues activation loop and C*α* helix. The radius of gyration (Rg) is defined as the mass weighted root mean square distance of atoms from their centre of mass. The competence, shape, and folding of the overall ALK structure at different time points during the trajectory can be seen in the plot of Rg (Figure 3(d)). During the first 5 ns, native and both the mutant complexes exhibited a similar pattern of Rg value, after which mutant complex F1174L showed a higher deviation with Rg score of ~2 nm. Despite the fact that mutant complex F1174L showed deviation from its starting conformation, both the mutants F1174L and R1275Q plateau around towards the end of the simulation. The number of NH bonds formed between crizotinib and protein models (native and mutant) during the MD simulation was also calculated (Figures 4(a), 4(b), and 4(c)). From our analysis, it is well revealed that native complex forms more number of NH bond with crizotinib with an average of ~3 NH bonds (Figure 4(c)), while the mutant complex F1174L exhibited less number of intermolecular NH bonds of an average of ~1-2 (Figures 4(a) and 4(c)). More intermolecular NH bonds in the native complex structure might help to maintain its rigidity while less tendency of the mutant involved in participating in hydrogen bonding with solvent makes it more flexible. Additional information on flexibility of ALK mutation was obtained by the analysis of the time-course of change in the secondary structures of native, F1174L, and R1275Q complexes during 20 ns MD simulations using the DSSP program (Figure 5). The native complex was stable over the 20 ns simulation and tends to have a conserved secondary structure during the simulation (Figure 5(a)), whereas the F1174L and R1275Q complexes showed subtle conformational fluctuations during the 20 ns simulation. Intermediate helix 3 (residue 1160–1173) of the native complex is disrupted (Figure 5(a)), and formation of the coil is observed in F1174L, and R1275Q complexes (Figures 5(b) and 5(c)). Rearrangements of secondary structural elements were observed between the residues (1264–1284), where turns and strands were lost, and additional helixes were formed in the structure. The C-terminal region of the protein becomes highly distorted due to the loss of C-terminal helix. To check the reliability of the simulations, we compared the noise in simulation data with the difference between native and mutant complex values.  Table 5 includes the mean values of the measured properties, their standard deviations, and the differences between the mean values of those properties in the native and mutant complexes.  Table 5 results indicate the differences between the mean values which are substantially greater than the standard deviation. This suggests that the observed change in ALK-crizotinib due to mutations is more reliable.

### 3.5. Principal Component Analysis

**Figure 6:**
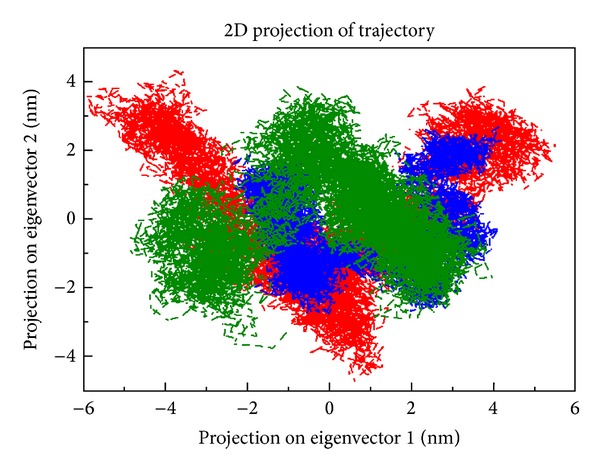
Projection of the motion of the protein in phase space along the first two principal eigenvectors at 300 K. Native type (green colour) versus F1174L (red colour) versus R1275Q (blue colour).

Eigen vectors with the largest associated eigenvalues define the essential subspace in which most of the protein dynamics occurs. On these projections, we can visualize the cluster of stable states. Two features are very apparent from these plots. Firstly the clusters are well defined in native than mutant complexes. Secondly, both the mutant complexes cover a larger region of phase space particularly along PC1 plane compared to that covered by native complex (Figure 6). Overall flexibility of the protein complexes was further examined by the trace of the diagonalized covariance matrix of the C*α* atomic positional fluctuations. Trace of the covariance matrix value of 11.395 nm^2^ was observed for the native complex and higher value of 18.259 nm^2^ and 13.942 was observed in ALK F1174L and ALK R1275Q mutant complexes. It is clear that mutant complex ALK F1174L behaved entirely in a different manner with trace of the covariance matrix value of 18.259 nm^2^ when compared to other protein complexes. This confirms the overall increased flexibility of mutant as compared to the native protein complex at 300 K.

### 3.6. Prediction of Posttranslational Modification Site

The prediction of posttranslational modifications sites in human ALK is presented in Table 6. These results show a high glycosylation and phosphorylation potential in human ALK. GPS 2.1 and NetPhos 2.0 predicted 41 sites where phosphorylation can occur. Similarly, analysis of glycosylation sites using NetNGlyc and NetOglyc predicted arginine residue at 15 different sites involved in N-linked glycosylation, and 4 threonine residues involved in O-linked glycosylation sites. From the predicted results, three phosphorylation sites at positions T1078, T1092, and T1604 were experimentally verified [55].

## 4. Discussion

Analyzing the effects of nsSNP derived from the disease on the functional property of a gene is advantageous to elucidate the most important function towards the pathogenesis. A detailed experimental analysis on the effect of nsSNPs in biological function is a daunting task. It is often time consuming and laborious to study the molecular basis of diseases like cancers, especially in cases where the number of mutations is very high. By contrast, detailed and useful information regarding the effect of nsSNP on protein structure and function can be readily obtained by* in silico* methods [2, 4]. Prioritizing the most interesting and likely pathogenic cases for further experimental analysis is another important application of the tested prediction methods. These methods make their prediction based on protein sequence and/or structural information as well as physiochemical property of amino acid for phenotype prediction. Especially integrating SNP information with protein 3D structure forms the basis of individual variability in drug response [11]. As a result, great strides have been attempted in understanding the structural details governing drug target interactions for recently approved therapeutics agents. Crystallographic studies convincingly demonstrate the important role of nsSNPs in protein flexibility and their efficacy for ligand binding. Expense and the extensive labor required to generate them have led to seek computational methods which can predict protein motions. Molecular dynamics simulation analysis developed in the late 1970s [56] seeks to overcome this limitation by using simple approximations based on Newtonian physics to simulate atomic motions, thus reducing the computational complexity. Previous studies have employed the structural information along with molecular dynamics to predict the impact of mutations in proteins [57–61] and protein drug complexes [62–66]. Indeed, quite numbers of studies have shown good concordance between computational and experimental measurements of macromolecular dynamics [57–59]. With constant improvements in both computer power and algorithm design, the attempts to identify the genetic determinants of different drug responses across a population to allow for the development of “individualized therapy” are promising; molecular dynamics simulations are likely to play an increasingly important role in the development of novel pharmacological therapeutics. Thus, we can expect that MD simulation would provide more reliable structural information on ALK* mutations*.

Application of* in silico* methods with different algorithms in pathogenicity or stability prediction is always debatable because of the discrepancy in their prediction scores even when analyzing the same variant. Therefore, no single method could be considered as the best and accurate for prediction of a functional SNP. Hence, a combination of methods based on evolutionary information and protein structure and/or functional parameters was used in order to increase the prediction accuracy. We utilized the experience gained by various groups to examine the effects of mutations and applicability of different* in silico* methods [67–72]. Our study gained significance by identifying the level of structural conformations changes with respect to the incorporation of deleterious mutations in* ALK* protein. By comparing the results of all the* in silico* prediction tools used in our study, two nsSNPs with positions F1174L and R1275Q were found to be highly deleterious. Our findings also revealed that the incorporation of different algorithms often serves as powerful tools for prioritizing candidate functional nsSNPs. Native-type phenylalanine 1174 lies at the C terminus $\overset{´}{\alpha }$ helix and its side chain contributes to the small well-packed hydrophobic core between $\overset{´}{\alpha }$C and the activation loop. The reduction in the size of the phenylalanine 1174 side chain will disturb the packing, which may affect the structural integrity of ALK tyrosine kinase domain. From the crystal structure, it is clear that phenylalanine 1174 also interacts with phenylalanine 1271 which forms a crucial part of DGF motif, the aspartate 1270 which is involved in the ATP binding pocket. Also, native-type residue forms salt bridge with glutamine 1160 and aspartate 1276. Replacing 1174 with mutant leucine residue, which is smaller, will create structural restraints thereby affecting the ALK stability. The R1275 side chain contributes to direct interaction between activation loop helix and the $\overset{´}{\alpha }$C helix, stabilizing the active conformation of active ALK tyrosine kinase domain conformation. Mutation R1275 with glutamine will disturb the activation loop thereby affecting the stability of protein. Mutant glutamine is smaller than native arginine, which may cause an empty space in the core of the protein. It would be speculative, yet interesting, to study the protein drug interaction and explicit solvent behavior of protein models in order to examine the difference in stability and dynamics behavior of native and mutant models. From docking analysis of ALK with crizotinib, it is well revealed that both the mutations perturbed the binding pocket quite significantly. The most notable change was seen in F1174L mutation which was well supported by an increase in binding energy and loss of hydrogen bond interaction with the neighboring protein when compared to the native protein. Many studies have mapped correlated motions and their perturbations due to mutation or ligand binding onto structures to link structural and thermodynamic changes [57, 65, 66]. In our study, a clear understanding of stability loss was seen in the RMSF, RMSD, SASA, and Rg plot which were also accompanied by less number of intermolecular NH bonds for F1174L when compared to native ALK and mutant R1275Q. Less intermolecular NH bonds in F1174L mutant structure might help to lose its rigidity and makes it more flexible. Further, from our PCA study it is clear that the mutant model F1174L has higher overall flexibility when compared to the native protein. This deviation might be due to disruption of secondary structure, which in turn affects the protein folding thereby decreasing the stability of protein. Therefore, we would suggest that F1174L mutation would have a great impact on protein function which was in good concordance with the results obtained by Mossé et al. [26]. We expect that the results from the current computational approach on ALK with suitable validation in near future will aid in understanding the effect of individual drug response and also has the capability to create personalized tools for the rapid diagnosis, prognosis and treatment of diseases.

## Supplementary Material

Table S1. nsSNPs analyzed by SIFT, PolyPhen 2.0, SNAP, SNPs&GO, I Mutant 3.0, PhD-SNP and ALIGN-GVGD in ALK gene.
Figure S1. Overall RMSD curve of native type and mutant ALK- crizotinib at 20 ns. Three independent simulations of each Protein-crizotinib complex. Time evolution of backbone RMSD is shown as a function of time for A) native type, B) F1174L and C) R1275Q.

## Figures and Tables

**Table 1:** Summary of *in silico* prediction methods, molecular docking, and molecular dynamics simulation approaches in nsSNP analysis.

*In silico* prediction methods based on sequence, structure information or combination of both	Website URL
SIFT BLink	http://sift.jcvi.org/www/SIFT_BLink_submit.html
PolyPhen 2	http://genetics.bwh.harvard.edu/pph2/
SNAP	https://www.rostlab.org/services/snap/
MutPred	http://mutpred.mutdb.org/
PANTHER	http://www.pantherdb.org/tools/csnpScoreForm.jsp
nsSNP Analyzer	http://snpanalyzer.uthsc.edu/
PhD-SNP	http://snps.biofold.org/phd-snp/phd-snp.html
Auto-Mute	http://proteins.gmu.edu/automute/
*I-Mutant Suite *	http://gpcr2.biocomp.unibo.it/cgi/predictors/I-Mutant3.0/I-Mutant3.0.cgi
Align GVGD	http://agvgd.iarc.fr/agvgd_input.php
Mutation Taster	http://www.mutationtaster.org/
Provean	http://provean.jcvi.org/index.php
Fathmm	http://fathmm.biocompute.org.uk/
SNPs3D	http://www.snps3d.org/
topoSNP	http://gila.bioengr.uic.edu/snp/toposnp/
CanPredict	http://research-public.gene.com/Research/genentech/canpredict/
LS-SNP/PDB	http://ls-snp.icm.jhu.edu/ls-snp-pdb/
KD4v	http://decrypthon.igbmc.fr/kd4v/cgi-bin/home
Parepro	http://www.mobioinfor.cn/parepro/
F-SNP	http://compbio.cs.queensu.ca/F-SNP/
***SNPeffect*** 4.0	http://snpeffect.switchlab.org/

Types of protein simulation	
Molecular Dynamics Simulation	Based on their interactions according to the equations of motion defined in classical (i.e., Newtonian) mechanics
Langevin Dynamics	Based on use of the Langevin equation as an alternative to Newton's second law
Brownian Dynamics	Diffusional analogue of MD carried out through the numerical integration of the Langevin equation
Monte Carlo	Stochastic approach under given thermodynamic conditions such as temperature and volume.
Simulated Annealing	Find the minimum energy configuration of a system
QM/MM	To study of biomolecular reaction mechanisms
Nondynamic Methods	Conformational Sampling, Principal Component Analysis

Tool For MD	
Gromacs	http://www.gromacs.org/
NAMD	http://www.ks.uiuc.edu/Research/namd/
AMBER	http://ambermd.org/
CHARMM	http://yuri.harvard.edu/

Tools for Modeling and Docking	
Chemical structure representations	Zinc Database, ChEMBL, Chemspider, Bingo, JChem for Excel, ChemDiff, Protein DataBank (PDB), Binding MOAD (Mother Of All Database), CREDO, TTD, STITCH, SMPDB
Molecular Modeling	CHARMM, GROMACS, Amber, SwissParam, Dundee PRODRG2 Server, PDB2PQR Server, SwissSideChain
Homology Modeling	Modeller, I-TASSER, LOMETS, SWISS-MODEL, SWISS-MODEL Repository, Robetta
Binding site prediction	MED-SuMo, CAVER, FINDSITE, sc-PDB, CASTp, Pocketome, 3DLigandSite, metaPocket, PocketAnnotate
Docking	Autodock, DOCK, GOLD, SwissDock, DockingServer, 1-Click Docking, COPICAT, Computer-Aided Drug-Design Platform using PyMOL, Haddock
Visualizing	Visual Molecular Dynamics, PyMOL, UCSF Chimera, Discovery studio

**Table 2:** SNPs in the regulatory region found to be functionally significant by FASTSNP.

SNP ID (rs)	Possible functional effects	Risk	Region
rs73920776	Promoter/regulatory region	1–3	5upstream
rs57277472	Promoter/regulatory region	1–3	5upstream
rs73920777	Promoter/regulatory region	1–3	5upstream
rs6727236	Promoter/regulatory region	1–3	5upstream
rs12151564	Promoter/regulatory region	1–3	5upstream
rs4666202	Promoter/regulatory region	1–3	5upstream
rs4666203	Promoter/regulatory region	1–3	5upstream
rs55793959	Promoter/regulatory region	1–3	5upstream
rs13404651	Promoter/regulatory region	1–3	5upstream
rs4666204	Promoter/regulatory region	1–3	5upstream
rs6731724	Promoter/regulatory region	1–3	5upstream

**Table 3:** Interactions of ALK native and mutant models with Crizotinib. AutoDock binding energy, nature of interaction, and participating residues are listed. Distance between drug atoms and residues involved in hydrogen bond is noted.

Protein model	Binding energy	Hydrogen bonding		Hydrophobic residues
	(Kcal/mol)	Residue	Distance (Å)	
Native	−9.21	Q 1197	2.80	Q 1146, 1196, A 1148, T 1258, L 1198, L L 1256, G 1202, A 1200.
		M 1199	1.97	

F1174L	−7.34	Q 1197	3.07	L 1122, A 1148, M 1196, M 1199, A 1200, G 1201, G 1202, R 1253, L 1256.

R1275Q	−8.07	M 1199	3.01	L 1122, A1148, Q 1197, L 1196, Q 1197 L 1198, A 1200, G 1202, L 1256, R 1253, D 1203, N 1254, D 1270, L 1150.

**Table 4:** Docking results of ALK with crizotinib using Patchdock.

RET	Score	ACE
Native	6894	−298.28
F1174L	5432	−144.17
R1275Q	5790	−176.08

**Table 5:** Calculated mean values for various properties, their standard deviations, and the differences between the mean values of native type and mutated ALK.

System	Mean	Standard deviation	Difference (Native-Mutant)
RMSD			
Native	0.244	0.01	
F1174L	0.33	0.08	0.086
R1275Q	0.285	0.03	0.041

RMSF			
Native	0.14	0.04	
F1174L	0.23	0.10	0.09
R1275Q	0.18	0.06	0.04

RG			
Native	1.90	0.180	
F1174L	1.96	0.193	0.06
R1275Q	1.93	0.189	0.03

SASA			
Native	76.2	1.42	
F1174L	82.9	1.91	6.7
R1275Q	81.8	1.86	5.6

**Table 6:** Predication of various phosphorylations and glycosylations sited in ALK.

Phosphorylation				Glycosylation	
NetPhos 2.0			GPS 2.0	Net N Glyc	NetOglyc
Serine	Threonine	Tyrosine	Tyrosine	Arginine	Threonine
31	505	240	90	169	1026
45	573	276	1059	244	1446
53	674	406	1278	324	1447
76	686	635	1282	411	1457
109	917	705	1283	445	
114	1151	734		563	
131	1307	772		571	
196	1363	**1078**		709	
205	1447	**1092**		808	
211	1512	1096		863	
225	1547	1507		864	
226	1607	**1604**		886	
				986	
				1115	
				1504	

Amino acid positions highlighted in bold were found to be experimentally verified.

## References

[1] Cline MS, Karchin R (2011). Using bioinformatics to predict the functional impact of SNVs. *Bioinformatics*.

[2] Karchin R (2009). Next generation tools for the annotation of human SNPs. *Briefings in Bioinformatics*.

[3] Wang Z, Moult J (2001). SNPs, protein structure, and disease. *Human Mutation*.

[4] Doss CGP, Sudandiradoss C, Rajasekaran R (2008). Applications of computational algorithm tools to identify functional SNPs. *Functional and Integrative Genomics*.

[5] Yue P, Li Z, Moult J (2005). Loss of protein structure stability as a major causative factor in monogenic disease. *Journal of Molecular Biology*.

[6] Joerger AC, Ang HC, Fersht AR (2006). Structural basis for understanding oncogenic p53 mutations and designing rescue drugs. *Proceedings of the National Academy of Sciences of the United States of America*.

[7] Bohl CE, Wu Z, Miller DD, Bell CE, Dalton JT (2007). Crystal structure of the T877A human androgen receptor ligand-binding domain complexed to cyproterone acetate provides insight for ligand-induced conformational changes and structure-based drug design. *Journal of Biological Chemistry*.

[8] Zhou T, Parillon L, Li F (2007). Crystal structure of the T315I mutant of AbI kinase. *Chemical Biology and Drug Design*.

[9] Kobayashi S, Boggon TJ, Dayaram T (2005). EGFR mutation and resistance of non-small-cell lung cancer to gefitinib. *The New England Journal of Medicine*.

[10] Daly AK (2010). Pharmacogenetics and human genetic polymorphisms. *Biochemical Journal*.

[11] Lahti JL, Tang GW, Capriotti E, Liu T, Altman RB (2012). Bioinformatics and variability in drug response: a protein structural perspective. *Journal of the Royal Society Interface*.

[12] Fernald GH, Capriotti E, Daneshjou R, Karczewski KJ, Altman RB (2011). Bioinformatics challenges for personalized medicine. *Bioinformatics*.

[13] Kumar P, Henikoff S, Ng PC (2009). Predicting the effects of coding non-synonymous variants on protein function using the SIFT algorithm. *Nature Protocols*.

[14] Adzhubei IA, Schmidt S, Peshkin L (2010). A method and server for predicting damaging missense mutations. *Nature Methods*.

[15] Capriotti E, Fariselli P, Rossi I, Casadio R (2008). A three-state prediction of single point mutations on protein stability changes. *BMC Bioinformatics*.

[16] Bromberg Y, Rost B (2007). SNAP: predict effect of non-synonymous polymorphisms on function. *Nucleic Acids Research*.

[17] Calabrese R, Capriotti E, Fariselli P, Martelli PL, Casadio R (2009). Functional annotations improve the predictive score of human disease-related mutations in proteins. *Human Mutation*.

[18] Capriotti E, Calabrese R, Casadio R (2006). Predicting the insurgence of human genetic diseases associated to single point protein mutations with support vector machines and evolutionary information. *Bioinformatics*.

[19] Tavtigian SV, Deffenbaugh AM, Yin L (2006). Comprehensive statistical study of 452 BRCA1 missense substitutions with classification of eight recurrent substitutions as neutral. *Journal of Medical Genetics*.

[20] Weigelt J (2010). Structural genomics-Impact on biomedicine and drug discovery. *Experimental Cell Research*.

[21] Palmer RH, Vernersson E, Grabbe C, Hallberg B (2009). Anaplastic lymphoma kinase: signalling in development and disease. *Biochemical Journal*.

[22] Stoica GE, Kuo A, Aigner A (2001). Identification of anaplastic lymphoma kinase as a receptor for the growth factor pleiotrophin. *Journal of Biological Chemistry*.

[23] Soda M, Choi YL, Enomoto M (2007). Identification of the transforming EML4-ALK fusion gene in non-small-cell lung cancer. *Nature*.

[24] Carén H, Abel F, Kogner P, Martinsson T (2008). High incidence of DNA mutations and gene amplifications of the ALK gene in advanced sporadic neuroblastoma tumours. *Biochemical Journal*.

[25] Jazii FR, Najafi Z, Malekzadeh R (2006). Identification of squamous cell carcinoma associated proteins by proteomics and loss of beta tropomyosin expression in esophageal cancer. *World Journal of Gastroenterology*.

[26] Mossé YP, Laudenslager M, Longo L (2008). Identification of ALK as a major familial neuroblastoma predisposition gene. *Nature*.

[27] Janoueix-Lerosey I, Lequin D, Brugières L (2008). Somatic and germline activating mutations of the ALK kinase receptor in neuroblastoma. *Nature*.

[28] George RE, Sanda T, Hanna M (2008). Activating mutations in ALK provide a therapeutic target in neuroblastoma. *Nature*.

[29] Carpenter EL, Mossé YP (2012). Targeting ALK in neuroblastoma-preclinical and clinical advancements. *Nature Reviews Clinical Oncology*.

[30] Kwak EL, Bang Y-J, Camidge DR (2010). Anaplastic lymphoma kinase inhibition in non-small-cell lung cancer. *The New England Journal of Medicine*.

[31] Choi YL, Soda M, Yamashita Y (2010). EML4-ALK mutations in lung cancer that confer resistance to ALK inhibitors. *The New England Journal of Medicine*.

[32] Yuan H-Y, Chiou J-J, Tseng W-H (2006). FASTSNP: an always up-to-date and extendable service for SNP function analysis and prioritization. *Nucleic Acids Research*.

[33] Guex N, Peitsch MC (1997). SWISS-MODEL and the Swiss-PdbViewer: an environment for comparative protein modeling. *Electrophoresis*.

[34] Hess B, Kutzner C, Van Der Spoel D, Lindahl E (2008). GRGMACS 4: algorithms for highly efficient, load-balanced, and scalable molecular simulation. *Journal of Chemical Theory and Computation*.

[35] Kutzner C, Van Der Spoel D, Fechner M (2007). Speeding up parallel GROMACS on high-latency networks. *Journal of Computational Chemistry*.

[36] Morris GM, Ruth H, Lindstrom W (2009). Software news and updates AutoDock4 and AutoDockTools4: automated docking with selective receptor flexibility. *Journal of Computational Chemistry*.

[37] Schneidman-Duhovny D, Inbar Y, Nussinov R, Wolfson HJ (2005). PatchDock and SymmDock: servers for rigid and symmetric docking. *Nucleic Acids Research*.

[38] Amberger J, Bocchini CA, Scott AF, Hamosh A (2009). McKusick’s online mendelian inheritance in man (OMIM). *Nucleic Acids Research*.

[39] Sherry ST, Ward M-H, Kholodov M (2001). DbSNP: the NCBI database of genetic variation. *Nucleic Acids Research*.

[40] Bairoch A, Apweiler R (1996). The SWISS-PROT protein sequence data bank and its new supplement TREMBL. *Nucleic Acids Research*.

[41] Berman HM, Westbrook J, Feng Z (2000). The Protein Data Bank. *Nucleic Acids Research*.

[42] Bava KA, Gromiha MM, Uedaira H, Kitajima K, Sarai A (2004). ProTherm, version 4.0: thermodynamic database for proteins and mutants. *Nucleic Acids Research*.

[43] Xue Y, Liu Z, Cao J (2011). GPS 2.1: enhanced prediction of kinase-specific phosphorylation sites with an algorithm of motif length selection. *Protein Engineering, Design and Selection*.

[44] Gupta R, Jung E NetNGlyc prediction of N-glycosylation sites in human proteins. software. http://www.cbs.dtu.dk/services/NetNGlyc/.

[45] Julenius K, Mølgaard A, Gupta R, Brunak S (2005). Prediction, conservation analysis, and structural characterization of mammalian mucin-type O-glycosylation sites. *Glycobiology*.

[46] Lee CC, Jia Y, Li N (2010). Crystal structure of the ALK (anaplastic lymphoma kinase) catalytic domain. *Biochemical Journal*.

[47] Ko J, Lee D, Park H, Coutsias EA, Lee J, Seok C (2011). The FALC-Loop web server for protein loop modeling. *Nucleic Acids Research*.

[48] Connolly ML (1983). Solvent-accessible surfaces of proteins and nucleic acids. *Science*.

[49] Connolly ML (1983). Analytical molecular surface calculation. *Journal of Applied Crystallography*.

[50] Zhang C, Vasmatzis G, Cornette JL, DeLisi C (1997). Determination of atomic desolvation energies from the structures of crystallized proteins. *Journal of Molecular Biology*.

[51] Van Gunsteren WF, Billeter SR, Eising AA, Hunenberger PH, Kruger P (1996). *Biomolecular Simulation: The GROMOS96 Manual and User Guide*.

[52] Schüttelkopf AW, Van Aalten DMF (2004). PRODRG: a tool for high-throughput crystallography of protein-ligand complexes. *Acta Crystallographica Section D: Biological Crystallography*.

[53] Amadei A, Linssen ABM, Berendsen HJC (1993). Essential dynamics of proteins. *Proteins: Structure, Function and Genetics*.

[54] Kabsch W, Sander C (1983). Dictionary of protein secondary structure: pattern recognition of hydrogen-bonded and geometrical features. *Biopolymers—Peptide Science Section*.

[55] Rush J, Moritz A, Lee KA (2005). Immunoaffinity profiling of tyrosine phosphorylation in cancer cells. *Nature Biotechnology*.

[56] McCammon JA, Gelin BR, Karplus M (1977). Dynamics of folded proteins. *Nature*.

[57] Zhang Z, Teng S, Wang L, Schwartz CE, Alexov E (2010). Computational analysis of missense mutations causing Snyder-Robinson syndrome. *Human Mutation*.

[58] Miteva MA, Brugge JM, Rosing J, Nicolaes GAF, Villoutreix BO (2004). Theoretical and Experimental Study of the D2194G mutation in the C2 domain of coagulation factor V. *Biophysical Journal*.

[59] Zhang Z, Norris J, Schwartz C, Alexov E (2011). In silico and in vitro investigations of the mutability of disease-causing missense mutation sites in spermine synthase. *PLoS ONE*.

[60] Witham S, Takano K, Schwartz C, Alexov E (2011). A missense mutation in CLIC2 associated with intellectual disability is predicted by in silico modeling to affect protein stability and dynamics. *Proteins: Structure, Function and Bioinformatics*.

[61] John AM, C. GPD, Ebenazer A (2013). p.Arg82Leu von Hippel-Lindau (VHL) gene mutation among three members of a family with familial bilateral pheochromocytoma in India: molecular analysis and in silico characterization. *PLoS ONE*.

[62] Yellapu NK, Kandlapalli K, Valasani KR, Sarma PV, Matcha B (2013). Structural variations of human glucokinase Glu256Lys in MODY2 condition using Molecular Dynamics Study. *Biotechnology Research International*.

[63] Dasgupta J, Sen U, Dattagupta JK (2003). In silico mutations and molecular dynamics studies on a winged bean chymotrypsin inhibitor protein. *Protein Engineering*.

[64] Nagasundaram N, George Priya Doss C (2013). Predicting the impact of single-nucleotide polymorphisms in CDK2-flavopiridol complex by molecular dynamics analysis. *Cell Biochemistry and Biophysics*.

[65] George Priya Doss C, Nagasundaram N, Chakraborty C, Chen L, Zhu H (2013). Extrapolating the effect of deleterious nsSNPs in the binding adaptability of flavopiridol with CDK7 protein: a molecular dynamics approach. *Human Genomics*.

[66] George Priya Doss C, Rajith B, Chakraborty C (2014). In silico profiling and structural insights of missense mutations in RET protein kinase domain by molecular dynamics and docking approach. *Molecular BioSystems*.

[67] Gray VE, Kukurba KR, Kumar S (2012). Performance of computational tools in evaluating the functional impact of laboratory-induced amino acid mutations. *Bioinformatics*.

[68] Gnad F, Baucom A, Mukhyala K, Manning G, Zhang Z (2013). Assessment of computational methods for predicting the effects of missense mutations in human cancers. *BMC Genomics*.

[69] Thusberg J, Olatubosun A, Vihinen M (2011). Performance of mutation pathogenicity prediction methods on missense variants. *Human Mutation*.

[70] Thusberg J, Vihinen M (2009). Pathogenic or not? and if so, then how? Studying the effects of missense mutations using bioinformatics methods. *Human Mutation*.

[71] Khan S, Vihinen M (2010). Performance of protein stability predictors. *Human Mutation*.

[72] Castellana S, Mazza T (2013). Congruency in the prediction of pathogenic missense mutations: state-of-the-art web-based tools. *Briefings in Bioinformatics*.

