# Supplementary material for: Integrating In Silico Prediction Methods, Molecular Docking, and Molecular Dynamics Simulation to Predict the Impact of ALK Missense Mutations in Structural Perspective
Source: Biomed Res Int. 2014 Jun 26;2014:895831. doi: 10.1155/2014/895831 (PMC4098886; doi:10.1155/2014/895831)
Supplement: Supplementary file 1 — Table S1. nsSNPs analyzed by SIFT, PolyPhen 2.0, SNAP, SNPs&GO, I Mutant 3.0, PhD-SNP and ALIGN-GVGD in ALK gene. Figure S1. Overall RMSD curve of native type and mutant ALK- crizotinib at 20 ns. Three independent simulations of each Protein-crizotinib complex. Time evolution of backbone RMSD is shown as a function of time for A) native type, B) F1174L and C) R1275Q. [file 895831.f1.doc]

**Table S1.** nsSNPs analyzed by SIFT, PolyPhen 2.0, SNAP, SNPs&GO, I Mutant 3.0, PhD-SNP and Align-GVGD in *ALK* gene

| **Reference IDs** | **Variants** | **SIFT** | **PolyPhen 2** | **SNAP** | **SNPs&GO** | **I Mutant 3** | **PhD- SNP** | **Align GVGD** |
| --- | --- | --- | --- | --- | --- | --- | --- | --- |
| rs201490095 | P36S | 0 | 0.0585 | Neutral | Neutral | 0.71 | Neutral | Class C0 |
| rs200212200 | P40L | 0 | 0.0469 | Neutral | Neutral | 0.16 | Neutral | Class C65 |
| rs78378278 | P43L | 0 | 0.0208 | Neutral | Neutral | 0.47 | Neutral | Class C65 |
| VAR_041477 | S90L | 0 | 0.0677 | Non-neutral | Disease | 0.52 | Neutral | Class C65 |
| rs201290745 | P117R | 0 | 0.0365 | Neutral | Disease | 1.43 | Neutral | Class C25 |
| rs74774946 | P157S | 0 | 0.86 | Neutral | Disease | 1.7 | Neutral | Class C65 |
| VAR_041478 | V163L | 0.05 | 0.86 | Neutral | Neutral | 1.3 | Neutral | Class C65 |
| rs62129830 | R179C | 0.03 | 0.165 | Non-neutral | Disease | 0.37 | Neutral | Class C25 |
| rs200564831 | E192G | 0 | 0.0469 | Non-neutral | Disease | 1 | Neutral | Class C25 |
| rs77677701 | V198M | 0.02 | 0.229 | Neutral | Neutral | 1.65 | Neutral | Class C2 |
| rs141093002 | A206T | 0.19 | 0.45 | Neutral | Neutral | 0.54 | Neutral | Class C65 |
| rs199584443 | G221R | 0.18 | 0.996 | Neutral | Neutral | 0.58 | Neutral | Class C15 |
| rs142120301 | T231R | 0 | 0.169 | Neutral | Neutral | 0.77 | Neutral | Class C45 |
| rs146281095 | F241L | 0.01 | 0.0854 | Neutral | Neutral | 2.42 | Neutral | Class C65 |
| rs138686378 | R259H | 0 | 0.0365 | Neutral | Disease | 1.52 | Neutral | Class C65 |
| rs140497106 | E265G | 0.06 | 0.117 | Non-neutral | Neutral | 1.25 | Neutral | Class C0 |
| rs143229596 | F270L | 0.04 | 0.0819 | Neutral | Disease | 1.66 | Disease | Class C55 |
| rs199703254 | P278L | 0.03 | 0.0849 | Neutral | Neutral | 0.57 | Neutral | Class C65 |
| rs112782816 | S289F | 0 | 0.0691 | Non-neutral | Neutral | 0.21 | Neutral | Class C0 |
| rs201750304 | R292C | 0.02 | 0.0681 | Neutral | Neutral | 0.02 | Neutral | Class C55 |
| rs149145987 | R292H | 0.31 | 0.723 | Neutral | Neutral | 1.04 | Neutral | Class C0 |
| rs199790669 | P307S | 0 | 0.0629 | Neutral | Neutral | 0.51 | Neutral | Class C15 |
| rs145681577 | A309T | 0.14 | 0.314 | Neutral | Neutral | 1.03 | Neutral | Class C25 |
| rs149837139 | R311C | 0.01 | 0.212 | Neutral | Neutral | 0.66 | Neutral | Class C65 |
| rs150966028 | R311H | 1 | 0.996 | Neutral | Neutral | 0.83 | Neutral | Class C65 |
| rs144135148 | S329F | 0 | 0.132 | Non-neutral | Disease | 0.5 | Neutral | Class C65 |
| rs147640694 | H344L | 0.02 | 0.0483 | Neutral | Neutral | 1.39 | Neutral | Class C65 |
| rs144030155 | P367R | 0.36 | 0.0456 | Neutral | Disease | 0.84 | Neutral | Class C65 |
| rs149968229 | A371T | 0.26 | 0.723 | Neutral | Neutral | 1.39 | Neutral | Class C55 |
| rs140928266 | K383M | 0.1 | 0.148 | Neutral | Neutral | 0.39 | Neutral | Class C65 |
| rs188859061 | R401Q | 0.24 | 0.102 | Neutral | Neutral | 1.88 | Neutral | Class C65 |
| rs147102592 | R412C | 0.01 | 0.0893 | Non-neutral | Disease | 1.41 | Neutral | Class C65 |
| rs111796753 | S426T | 0.87 | 0.723 | Neutral | Neutral | 0.88 | Neutral | Class C0 |
| rs199970780 | L451V | 0.1 | 0.723 | Neutral | Neutral | 0.93 | Neutral | Class C65 |
| rs55706535 | G464R | 0 | 0.0781 | Non-neutral | Neutral | 0.59 | Neutral | Class C65 |
| VAR_041480 | V476A | 0.53 | 0.996 | Neutral | Neutral | 1.09 | Neutral | Class C65 |
| rs267599336 | S495L | 0.03 | 0.287 | Non-neutral | Neutral | 0.64 | Neutral | Class C65 |
| rs201449759 | R510W | 0.12 | 0.996 | Non-neutral | Neutral | 0.01 | Neutral | Class C65 |
| rs202106175 | V526D | 0.66 | 0.996 | Non-neutral | Neutral | 2.91 | Neutral | Class C25 |
| rs147033307 | S545N | 1 | 0.996 | Neutral | Neutral | 0.7 | Neutral | Class C65 |
| rs200364883 | R557G | 0.18 | 0.176 | Non-neutral | Disease | 1.18 | Neutral | Class C55 |
| rs200468507 | R557H | 0.35 | 0.307 | Non-neutral | Disease | 0.97 | Neutral | Class C65 |
| VAR_041481 | L560F | 0.51 | 0.314 | Non-neutral | Neutral | 0.39 | Neutral | NA |
| rs75158395 | A586P | 0.13 | 0.996 | Neutral | Disease | 0.78 | Neutral | Class C65 |
| rs61744521 | A613P | 0 | 0.147 | Non-neutral | Disease | 2.04 | Disease | Class C65 |
| rs144453491 | G640R | 0.01 | 0.197 | Non-neutral | Neutral | 2.12 | Neutral | Class C45 |
| rs116202066 | T648I | 0.35 | 0.276 | Neutral | Neutral | 1.45 | Neutral | Class C65 |
| rs267599335 | L655R | 0.05 | 0.249 | Neutral | Neutral | 0.92 | Neutral | Class C65 |
| rs138534542 | P666L | 0.07 | 0.86 | Neutral | Neutral | 0.68 | Neutral | Class C45 |
| rs149640902 | N669S | 0.2 | 0.287 | Neutral | Neutral | 0.27 | Neutral | Class C65 |
| rs145780832 | P671S | 0.51 | 0.996 | Neutral | Neutral | 0.84 | Neutral | Class C65 |
| VAR_041482 | T680I | 0.01 | 0.218 | Non-neutral | Neutral | 0.36 | Neutral | Class C45 |
| VAR_041483 | A704T | 0 | 0.307 | Non-neutral | Neutral | 0.83 | Neutral | Class C15 |
| rs78723472 | V712M | 0.01 | 0.0581 | Neutral | Neutral | 1.14 | Disease | Class C15 |
| rs147858673 | E717K | 0.06 | 0.165 | Neutral | Neutral | 0.83 | Neutral | Class C15 |
| rs140527448 | G722A | 0 | 0.0138 | Neutral | Disease | 0.91 | Neutral | Class C0 |
| rs187200776 | R753L | 0.01 | 0.138 | Non-neutral | Disease | 0.9 | Disease | Class C55 |
| rs199691702 | V759M | 0.05 | 0.263 | Neutral | Neutral | 2.03 | Neutral | Class C15 |
| rs80227749 | R806C | 0.01 | 0.222 | Non-neutral | Disease | 0.44 | Neutral | Class C65 |
| rs200486099 | A815S | 0.02 | 0.133 | Neutral | Neutral | 0.38 | Neutral | Class C65 |
| rs201590062 | G821R | 0 | 0.0692 | Non-neutral | Disease | 0.04 | Disease | Class C65 |
| rs142641078 | V836L | 0.37 | 0.201 | Neutral | Neutral | 1.51 | Neutral | Class C35 |
| rs200563480 | G850R | 0.28 | 0.86 | Non-neutral | Neutral | 0.87 | Neutral | Class C55 |
| rs189078025 | D854N | 0 | 0.167 | Neutral | Disease | 0.61 | Neutral | Class C65 |
| rs61754865 | E859D | 0.08 | 0.0581 | Neutral | Neutral | 0.61 | Neutral | Class C0 |
| VAR_061288 | L868Q | 0.1 | 0.723 | Neutral | Neutral | 1.37 | Neutral | Class C15 |
| rs148001139 | G875R | 0.03 | 0.0403 | Non-neutral | Disease | 0.07 | Neutral | Class C45 |
| VAR_041484 | A877S | 0 | 0.0581 | Non-neutral | Neutral | 0.15 | Neutral | Class C65 |
| rs79530637 | G923E | 0 | 0.00692 | Non-neutral | Disease | 1.06 | Neutral | Class C65 |
| rs78086548 | G924S | 0.02 | 0.0138 | Neutral | Disease | 0.79 | Neutral | Class C0 |
| rs75861155 | G924D | 0.1 | 0.0138 | Neutral | Neutral | 0.62 | Neutral | Class C0 |
| rs76870042 | G927R | 0 | 0.0937 | Non-neutral | Neutral | 0.4 | Neutral | Class C0 |
| rs150344432 | E994K | 0.3 | 0.111 | Neutral | Neutral | 0.55 | Neutral | Class C15 |
| VAR_041485 | T1012M | 0.28 | 0.189 | Non-neutral | Neutral | 0.4 | Neutral | Class C65 |
| rs76742576 | V1019I | 0.07 | 0.134 | Neutral | Neutral | 0.69 | Neutral | Class C35 |
| rs140733978 | P1027L | 0.08 | 0.272 | Neutral | Neutral | 1.37 | Neutral | Class C65 |
| rs200992945 | H1030P | 0.31 | 0.175 | Neutral | Neutral | 0.87 | Neutral | Class C65 |
| rs139365887 | L1033F | 0.23 | 0.254 | Neutral | NA | 0.38 | Neutral | Class C55 |
| rs200080181 | V1039M | 0.01 | 0.0665 | Non-neutral | Neutral | 4.01 | Neutral | Class C65 |
| rs200641396 | V1045M | 0.07 | 0.314 | Neutral | Neutral | 4.34 | Neutral | Class C65 |
| rs143452915 | V1049I | 0.72 | 0.86 | Neutral | NA | 1.41 | Neutral | Class C25 |
| rs72852032 | R1061Q | 0.05 | 0.083 | Neutral | NA | 0.47 | Disease | Class C15 |
| rs138178848 | Q1070H | 0.23 | 0.154 | Neutral | Neutral | 1.21 | Neutral | Class C25 |
| rs138589984 | S1086L | 0.05 | 0.0346 | Neutral | Neutral | 0.5 | Neutral | Class C35 |
| rs113994090 | T1087I | 0 | 0.138 | Neutral | NA | 1.33 | Neutral | Class C35 |
| VAR_063850 | D1091N | 0.34 | 0.0423 | Neutral | Neutral | 1.32 | Neutral | Class C15 |
| rs140606509 | E1110D | 0.07 | 0.0442 | Neutral | NA | 0.31 | Disease | Class C35 |
| rs199987354 | R1113Q | 0 | 0 | Neutral | NA | -1.53 | Disease | Class C55 |
| VAR_041486 | G1121D | 0.02 | 0.0757 | Non-neutral | NA | 1.33 | Disease | Class C55 |
| VAR_063851 | G1128A | 0 | 0.0692 | Non-neutral | Disease | 0.3 | Disease | Class C35 |
| rs76805758 | V1149G | 0 | 0.0403 | Neutral | NA | 4.22 | Disease | Class C0 |
| rs200916982 | K1150E | 0 | 0.997 | Non-neutral | Neutral | 0.56 | Disease | Class C15 |
| VAR_063852 | T1151M | 0 | 0 | Neutral | NA | 0.1 | Neutral | Class C65 |
| rs145194836 | E1161K | 0.08 | 0.0138 | Neutral | Neutral | 0.11 | Disease | Class C65 |
| VAR_063853 | M1166R | 0.05 | 0 | Neutral | Neutral | 1.46 | Neutral | Class C65 |
| VAR_063854 | I1171N | 0 | 1 | Neutral | NA | -0.75 | Neutral | Class C0 |
| VAR_063856 | F1174I | 0 | 1 | Neutral | NA | 0.03 | Neutral | Class C65 |
| VAR_063858 | F1174V | 0 | 1 | Neutral | NA | -0.35 | Neutral | Class C65 |
| VAR_063855 | F1174C | 0 | 1 | Neutral | Neutral | -0.51 | Neutral | Class C25 |
| **VAR_063857** | **F1174 L** | **0** | **1** | **Non-neutral** | **Disease** | **-1.34** | **Disease** | **Class C65** |
| rs56315533 | R1181C | 0 | 1 | Non-neutral | Neutral | -0.57 | Neutral | Class C65 |
| rs149995444 | V1185L | 0.07 | 0.777 | Neutral | NA | 0.3 | NA | Class C65 |
| VAR_063859 | R1192P | 0.03 | 1 | Neutral | Disease | -1.88 | Disease | Class C0 |
| rs200585833 | A1200V | 0.1 | 0.991 | Neutral | NA | -0.38 | Disease | Class C25 |
| rs143790259 | R1212H | 0 | 1 | Neutral | Neutral | -0.92 | Disease | Class C55 |
| rs200110351 | R1231Q | 0.34 | 0.027 | Neutral | NA | -2.18 | Disease | Class C0 |
| VAR_063860 | A1234T | 0.02 | 1 | Non-neutral | NA | 0.8 | NA | Class C0 |
| VAR_063861 | F1245C | 0 | 1 | Non-neutral | Disease | -0.59 | Neutral | Class C0 |
| VAR_063862 | F1245 V | 0 | 1 | Non-neutral | NA | 0.15 | Neutral | Class C65 |
| VAR_063863 | I1250T | 0 | 1 | Neutral | NA | -2.41 | Neutral | Class C65 |
| VAR_041487 | A1274T | 0.04 | 1 | Neutral | Neutral | -0.79 | Disease | Class C55 |
| **VAR_063865** | **R1275Q** | **0** | **1** | **Non-neutral** | **Disease** | **-1.71** | **Disease** | **Class C65** |
| VAR_063864 | R1275 L | 0 | 1 | Non-neutral | NA | -0.55 | Disease | Class C25 |
| VAR_063866 | Y1278S | 0 | 0.998 | Neutral | Disease | 0.8 | NA | Class C55 |
| rs74716434 | A1280V | 0.1 | 1 | Non-neutral | NA | -1.77 | Neutral | Class C45 |
| rs13427480 | R1284K | 0.03 | 1 | Neutral | NA | -1.05 | Disease | Class C35 |
| VAR_041488 | M1328L | 0.09 | 1 | Non-neutral | Neutral | -0.13 | Neutral | Class C65 |
| VAR_055987 | F1376S | 0 | 1 | Non-neutral | NA | -0.43 | Neutral | Class C15 |
| rs200902932 | Q1388H | 0.1 | 0.793 | Neutral | NA | -0.60 | Disease | Class C15 |
| rs201768549 | A1396T | 0.09 | 0.826 | Neutral | Disease | -0.54 | Disease | Class C35 |
| rs143647372 | E1400D | 0.21 | 0.103 | Neutral | NA | -0.79 | Disease | Class C55 |
| rs145600484 | E1408L | 0.01 | 1 | Non-neutral | NA | -0.38 | Disease | Class C65 |
| rs139086136 | E1409D | 0.54 | 0.996 | Neutral | Disease | -0.77 | Disease | Class C65 |
| rs77762612 | R1414K | 0.12 | 0.986 | Non-neutral | NA | 0.29 | Disease | Class C65 |
| VAR_041489 | K1416N | 0.35 | 0.001 | Neutral | NA | -2.02 | Disease | Class C55 |
| VAR_041490 | E1419K | 0.13 | 0.863 | Non-neutral | Disease | -0.59 | Disease | Class C25 |
| rs200470125 | G1420E | 0.22 | 0.986 | Neutral | NA | -1.82 | Neutral | Class C65 |
| VAR_041491 | Q1429R | 0.4 | 0.793 | Non-neutral | NA | -0.11 | Neutral | Class C0 |
| rs201129468 | R1436C | 0.09 | 0.003 | Non-neutral | NA | -1.24 | Neutral | Class C25 |
| rs80004824 | P1442A | 0.41 | 0.793 | Neutral | Neutral | -0.58 | Neutral | Class C15 |
| rs111323577 | P1415L | 0.29 | 0.998 | Non-neutral | NA | -0.53 | Neutral | Class C0 |
| rs112657055 | T1457I | 0.2 | 0.032 | Non-neutral | Disease | 0.39 | Neutral | Class C25 |
| rs149306495 | E1460G | 0.02 | 0.001 | Neutral | NA | -0.52 | Neutral | Class C15 |
| VAR_031042 | I1461V | 0.6 | 0.000 | Neutral | NA | -0.61 | Neutral | Class C65 |
| rs201759867 | V1471M | 0.18 | 0.075 | Neutral | Disease | 0.12 | Neutral | Class C25 |
| rs141010693 | M1478T | 0.75 | 0.144 | Non-neutral | NA | -0.32 | Neutral | Class C35 |
| VAR_031043 | K1491R | 0.34 | 0.000 | Non-neutral | Neutral | -0.54 | Disease | Class C0 |
| rs199940609 | G1494R | 0.1 | 0.377 | Non-neutral | NA | -1.30 | Disease | Class C25 |
| rs75681494 | T1512I | 0.1 | 0.102 | Non-neutral | NA | -1.22 | Neutral | Class C15 |
| rs148138515 | E1513D | 0.08 | 0.996 | Neutral | Disease | -0.13 | Neutral | Class C0 |
| VAR_031044 | D1529E | 1 | 0.000 | Non-neutral | NA | -0.74 | Neutral | Class C35 |
| rs141242925 | R1530K | 0.69 | 0.055 | Non-neutral | NA | -0.84 | Disease | Class C0 |
| rs139185626 | G1534E | 0.2 | 0.905 | Non-neutral | NA | -1.38 | Disease | Class C25 |
| rs78868998 | G1548E | 0.1 | 0.181 | Non-neutral | Disease | -0.62 | Neutral | Class C25 |
| rs199646095 | R1549I | 0.1 | 0.996 | Non-neutral | NA | 0.42 | Neutral | Class C25 |
| rs148351049 | R1575C | 0 | 1 | Non-neutral | Neutral | -0.96 | Disease | Class C65 |
| rs200829376 | F1577V | 0.01 | 0.986 | Non-neutral | NA | 0.51 | Neutral | Class C55 |
| rs190639819 | G1590S | 0.2 | 0.959 | Neutral | Neutral | -0.67 | Disease | Class C0 |
| VAR_055988 | P1599H | 0 | 0.996 | Non-neutral | NA | NA | NA | Class C0 |

**nsSNP IDs highlighted in bold are predicted to be deleterious by all tools, NA- not applicable**

**Figure S1**
